# Pregnancy-associated *Enterococcus faecalis* aortic valve endocarditis secondary to obstructive uropathy: a case report

**DOI:** 10.1093/ehjcr/ytag387

**Published:** 2026-05-29

**Authors:** Fenghan Zhang, Shazia Aziz, Shivarpit Dua, Bryce Grohol, Ijeoma Ezeife

**Affiliations:** Department of Internal Medicine, ChristianaCare Health System, 4755 Ogletown-Stanton Road, Newark, DE 19718, USA; Department of Internal Medicine, ChristianaCare Health System, 4755 Ogletown-Stanton Road, Newark, DE 19718, USA; Department of Cardiology, ChristianaCare Health System, 4755 Ogletown-Stanton Road, Newark, DE 19718, USA; Department of Internal Medicine, ChristianaCare Health System, 4755 Ogletown-Stanton Road, Newark, DE 19718, USA; Department of Internal Medicine, ChristianaCare Health System, 4755 Ogletown-Stanton Road, Newark, DE 19718, USA; Department of Internal Medicine, ChristianaCare Health System, 4755 Ogletown-Stanton Road, Newark, DE 19718, USA; Department of Cardiology, ChristianaCare Health System, 4755 Ogletown-Stanton Road, Newark, DE 19718, USA

**Keywords:** Case report, Infective endocarditis, Aortic regurgitation, Valvular heart disease, Cardio-obstetrics

## Abstract

**Background:**

Infective endocarditis during pregnancy is rare but associated with significant maternal and foetal morbidity, particularly in the setting of *Enterococcus faecalis* bacteraemia from genitourinary sources.

**Case summary:**

A 28-year-old woman at 32 weeks’ gestation with bilateral nephrolithiasis and indwelling nephrostomy tubes presented with sepsis. Blood cultures grew *E. faecalis*. Transoesophageal echocardiography revealed a mobile aortic valve vegetation with progressive severe aortic regurgitation. A multidisciplinary team coordinated staged management, including antimicrobial therapy, genitourinary source control, planned delivery, and postpartum mechanical aortic valve replacement.

**Discussion:**

This case highlights the strong association between *E. faecalis* bacteraemia and infective endocarditis, the importance of early echocardiography, and the need for individualized, multidisciplinary decision-making in pregnancy.

Learning points

- *Enterococcus faecalis* bacteraemia should prompt early evaluation for infective endocarditis, even in young pregnant patients.
- Multidisciplinary, staged management is essential when severe valvular disease complicates pregnancy.

## Introduction

Infective endocarditis (IE) during pregnancy is rare but associated with substantial maternal and foetal morbidity and mortality.^1^  *Enterococcus faecalis* bacteraemia carries a significant risk of underlying valvular infection, particularly in patients with genitourinary pathology.^1^ Management is challenging because progressive valvular dysfunction must be balanced against maternal haemodynamic stability, foetal maturity, and the timing of definitive surgical intervention. We report a case of pregnancy-associated *E. faecalis* aortic valve endocarditis secondary to obstructive uropathy complicated by progressive severe aortic regurgitation (AR) requiring staged multidisciplinary management and postpartum surgical aortic valve replacement.

## Summary figure

**Table:** 

Clinical timeline	Cardiac events	Obstetric/genitourinary events
32 weeks’ gestation	Presentation with sepsis; blood cultures positive	Admission to obstetric triage
32 weeks	Transthoracic echocardiography (TTE): possible aortic valve lesion	
32–33 weeks	Transoesophageal echocardiography (TEE): aortic valve vegetation with moderate AR	
33 weeks’ gestation	Multidisciplinary planning	Induction of labour in cardiac intensive care unit (ICU); vaginal delivery
Peripartum hospitalization	i.v. antibiotics initiated (*E. faecalis*)	Computed tomography (CT) abdomen: nephrolithiasis with nephrostomy tubes
Early postpartum	Repeat TTE: persistent vegetation; worsening AR	Stone management planning
Postpartum Month 1	TTE: progression to severe AR	Percutaneous nephrolithotomy (PCNL) and ureteral procedures for source control
Postpartum Month 2	TEE/ computed tomography angiography (CTA): residual vegetation; severe AR	Continued genetal urinary (GU) interventions
Postpartum Month 3	Surgical aortic valve replacement (mechanical valve)	
Postoperative course	Stable recovery	Right ureteral stent placement

## Case presentation

A 28-year-old woman (G4P2A1) at 32 weeks’ gestation presented with fever, chills, myalgias, and dyspnoea. Vital signs were notable for a temperature of 101.4°F and tachycardia (116 beats per minute). Physical examination revealed tachycardia and a gravid uterus without signs of heart failure or embolic phenomena.

Her past medical history was notable for bilateral nephrolithiasis complicated by obstructive uropathy requiring bilateral percutaneous nephrostomy tubes. She had recurrent urinary tract infections managed with nitrofurantoin suppression and a prior hospitalization for urosepsis related to nephrolithiasis.

The differential diagnosis for her presentation with fever, dyspnoea, and systemic symptoms in late pregnancy included urosepsis related to obstructive nephrolithiasis, pneumonia, pulmonary embolism, and infective endocarditis. Given her history of nephrolithiasis with indwelling nephrostomy tubes and recurrent urinary tract infections, a genitourinary source of infection was initially suspected.

**Figure 1:**
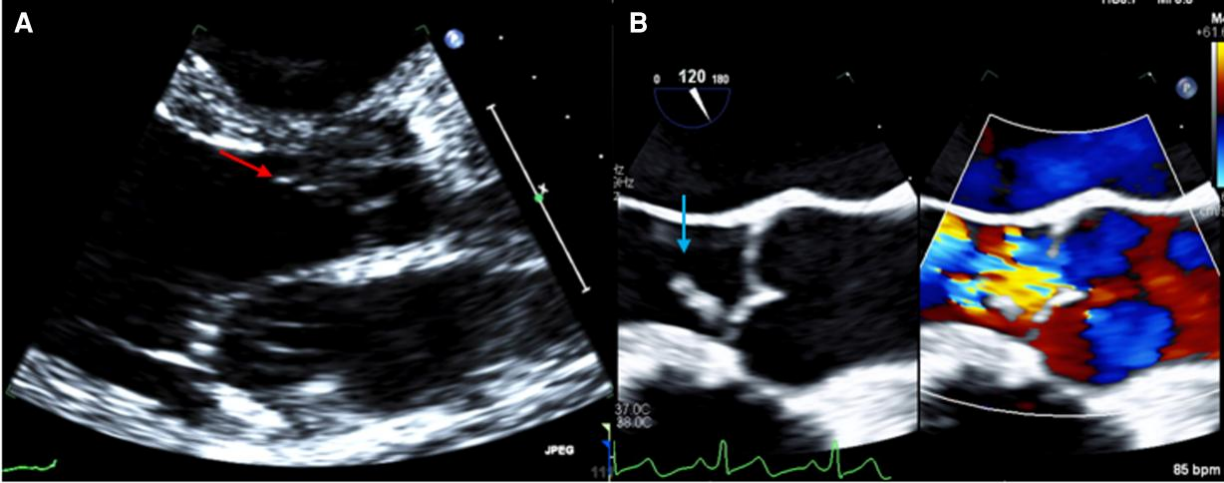
Echocardiographic findings in aortic valve endocarditis. (*A*) Initial transthoracic echocardiography (parasternal long-axis view) demonstrating a possible echogenic lesion on the aortic valve suggestive of vegetation (arrow). (*B*) Transoesophageal echocardiography (mid-oesophageal long-axis view) demonstrating a mobile aortic valve vegetation (arrow) with associated colour Doppler evidence of aortic regurgitation.

Laboratory studies demonstrated leukocyturia and bacteriuria. Blood cultures grew *E. faecalis* within 24 h. Transthoracic echocardiography (*Figure 1A*) demonstrated a possible aortic valve echodensity, and TEE (*Figure 1B*) confirmed a 1.1 × 0.4 cm mobile vegetation on the right coronary cusp with moderate, later severe, AR. Serial imaging demonstrated progressive left ventricular dilation.

Following the identification of *E. faecalis* bacteraemia, antimicrobial therapy was narrowed to intravenous ampicillin and ceftriaxone for synergistic treatment in accordance with guideline recommendations for enterococcal infective endocarditis. Given the strong association between *E. faecalis* bacteraemia and infective endocarditis, echocardiographic evaluation was performed, prompting TEE for definitive diagnosis (*Figure 1*).

**Figure 2:**
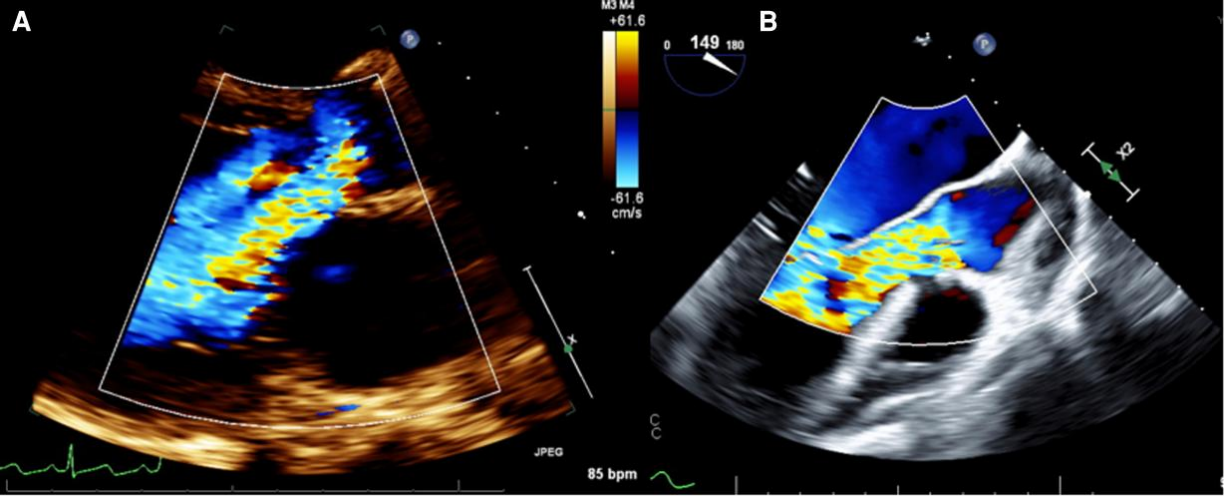
Echocardiographic demonstration of progressive aortic regurgitation. (*A*) Follow-up transthoracic echocardiography (parasternal long-axis view) demonstrating worsening aortic regurgitation. Pulsed-wave Doppler of the abdominal aorta shows prominent holodiastolic flow reversal, consistent with severe aortic regurgitation. (*B*) Transoesophageal echocardiography (mid-oesophageal long-axis view) demonstrating severe aortic regurgitation on colour Doppler.

Serial transthoracic and transoesophageal echocardiograms (*Figure 2A* and *B*) were performed throughout hospitalization to monitor vegetation morphology, valvular function, and ventricular performance. These studies demonstrated progression to severe AR with persistent vegetation, while left ventricular size and systolic function remained preserved without clinical or echocardiographic evidence of heart failure.

A multidisciplinary team, including cardiology, cardiothoracic surgery, maternal–foetal medicine, infectious diseases, urology, interventional radiology, psychiatry, and neonatology, convened to determine the optimal timing of intervention, balancing maternal cardiac risk, foetal maturity, and infection control. Although the vegetation size met Class IIb criteria for early surgical intervention, the absence of heart failure, preserved ventricular function, and pregnancy-associated operative risk supported a staged management approach.

Antenatal corticosteroids were administered, and planned induction of labour was performed in the cardiac intensive care unit to mitigate risks associated with intrapartum haemodynamic changes. Spontaneous vaginal delivery occurred at 33 weeks’ and 5 days’ gestation and was uncomplicated. Postpartum, repeat echocardiography continued to demonstrate severe AR with residual valvular vegetation, raising concern for ongoing embolic risk and progressive valvular dysfunction despite appropriate antimicrobial therapy.

In parallel, definitive genitourinary source control was pursued through staged nephrostomy tube management, ureteral stent placement, extracorporeal shockwave lithotripsy, and partial percutaneous nephrolithotomy. Stone analysis demonstrated calcium phosphate and calcium oxalate monohydrate composition.

**Figure 3:**
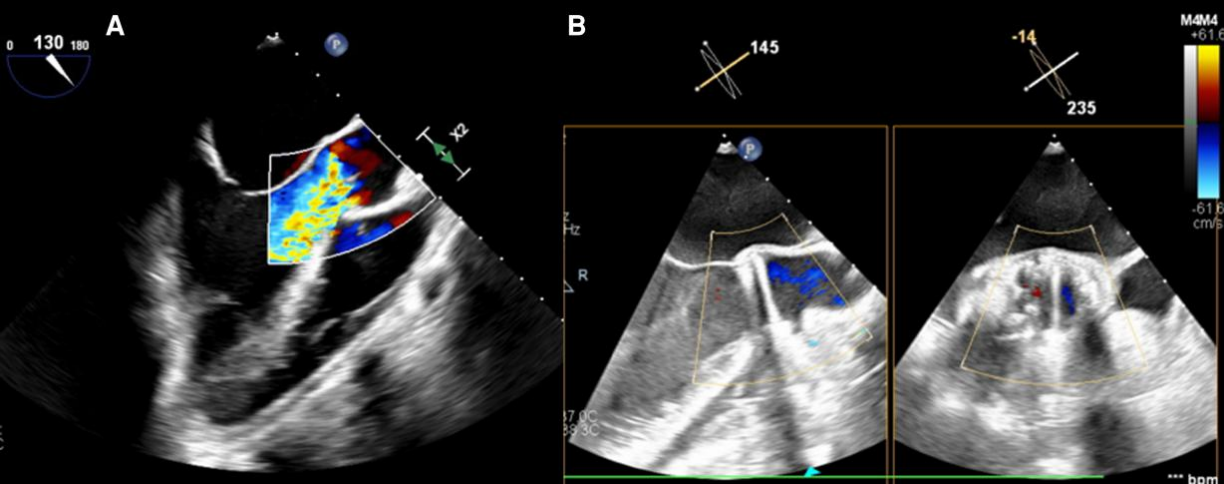
Preoperative severe aortic regurgitation and postoperative mechanical aortic valve replacement. (*A*) Preoperative transoesophageal echocardiography demonstrating severe aortic regurgitation on colour Doppler, consistent with progression of valvular dysfunction. (*B*) Postoperative transthoracic echocardiography demonstrating a mechanical aortic valve prosthesis following surgical aortic valve replacement.

Following genitourinary source control and clinical optimization, preoperative TEE (*Figure 3A*) confirmed severe AR from an anteriorly directed jet between the noncoronary and right coronary cusps. The patient subsequently underwent surgical aortic valve replacement (SAVR) with a 23 mm mechanical prosthesis. Postoperative TTE (*Figure 3B*) demonstrated a well-functioning mechanical valve with resolution of regurgitation, and a right double-J ureteral stent was placed for ureteral obstruction.

Postoperatively, the patient remained haemodynamically stable without the need for vasoactive support. Her hospital course was notable for acute blood loss anaemia (nadir haemoglobin 7.8 g/dL), managed conservatively without transfusion. She completed a 6-week course of intravenous ampicillin and ceftriaxone with no recurrence of fever or bacteraemia and was initiated on warfarin anticoagulation with a target international normalized ratio (INR) of 2.0–2.5.

**Figure 4:**
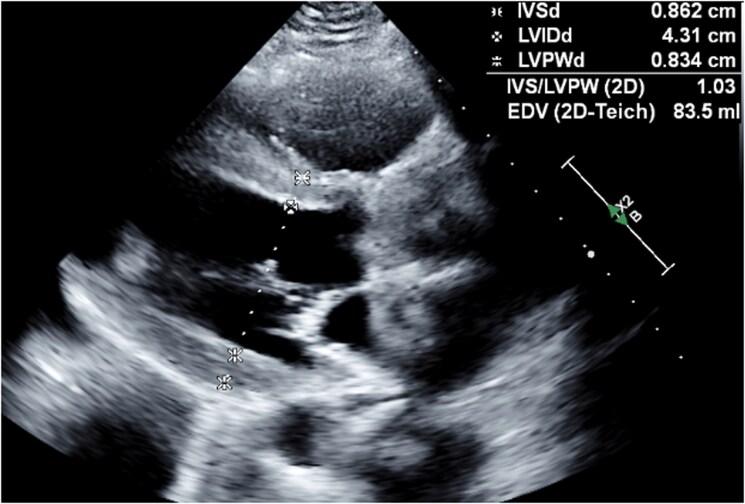
Follow-up transthoracic echocardiography (parasternal long-axis view) demonstrating normal left ventricular size and a normally functioning mechanical aortic valve prosthesis.

At 1-year follow-up, TTE (*Figure 4*) demonstrated stable function of the mechanical aortic valve prosthesis with no stenosis or significant regurgitation. No clinical embolic events occurred during hospitalization or follow-up. The patient reported no symptoms of dyspnoea or orthopnoea and continued close follow-up with cardiology, infectious diseases, and urology, with no evidence of recurrent infection or valve-related complications.

## Discussion

IE during pregnancy is rare but associated with significant maternal and foetal morbidity.^1–3^ Physiologic haemodynamic changes in pregnancy—including increased plasma volume, elevated cardiac output, and reduced systemic vascular resistance—can both mask early valvular dysfunction and amplify clinical deterioration once significant regurgitation develops. This case illustrates the complex interplay between *enterococcal* bacteraemia, progressive AR, and multidisciplinary decision-making in the peripartum period.

*Enterococcus faecalis* bacteraemia carries a high prevalence of underlying IE, particularly in patients with genitourinary pathology.^4,5^ Both the 2023 European Society of Cardiology (ESC) Guidelines for the Management of Endocarditis and prior American Heart Association (AHA) scientific statements recommend systematic echocardiographic evaluation in patients with *E. faecalis* bacteraemia due to the substantial risk of valvular involvement.^6,7^ In this patient, obstructive nephrolithiasis with indwelling nephrostomy tubes likely served as the source of bacteraemia, and early TEE was pivotal in confirming aortic valve vegetation.

Management of left-sided IE during pregnancy requires individualized, multidisciplinary coordination among cardiology, maternal–foetal medicine, cardiothoracic surgery, infectious disease, and urology teams. According to the 2023 ESC Endocarditis Guidelines and the 2020 American College of Cardiology (ACC)/AHA Guideline for the Management of Patients With Valvular Heart Disease, surgical intervention in native valve IE is indicated for heart failure due to severe valvular regurgitation, uncontrolled infection, or prevention of embolic complications in select high-risk patients.^6,8^ Although the vegetation size in this case met Class IIb criteria for early surgery, the absence of overt heart failure and preserved ventricular function allowed for initial medical management and continuation of pregnancy under close surveillance. Delivery was performed in a cardiac intensive care setting to mitigate the haemodynamic stress of labour in the context of evolving AR.

Despite appropriate antimicrobial therapy with ampicillin and ceftriaxone and aggressive genitourinary source control, serial imaging demonstrated progression of AR from moderate to severe. Ampicillin plus ceftriaxone has been shown to be an effective and guideline-supported regimen for the treatment of *E. faecalis* infective endocarditis.^9^ The 2020 ACC/AHA Valvular Heart Disease Guidelines recommend surgical intervention for severe AR in symptomatic patients or in those with evidence of progressive ventricular remodelling.^8^ Given the progression of AR from moderate to severe on serial imaging and the associated risk of decompensation, definitive SAVR was pursued postpartum. Surgical intervention in IE has been shown to improve outcomes in selected patients with severe valvular disease.^10^ Valve selection was individualized after shared decision-making, balancing durability against the implications of long-term anticoagulation.

This case highlights several key principles: (i) *E. faecalis* bacteraemia warrants urgent echocardiographic evaluation; (ii) multidisciplinary management is essential in peripartum IE; (iii) serial imaging is critical in detecting progression of AR; and (iv) definitive source control is central to reducing reinfection risk prior to prosthetic valve implantation. Timely diagnosis, coordinated multidisciplinary care, and adherence to guideline-directed management resulted in favourable maternal and foetal outcomes.

## Data Availability

The data underlying this article will be shared on reasonable request for corresponding author.

## Lead author biography

Fenghan Zhang is an internal medicine resident at ChristianaCare in Delaware, USA.

## Notes

**Consent:** The authors have obtained consent from the patient to publish this case report, including images, in accordance with the COPE guidelines
